# Aiming the magic bullet: targeted delivery of imaging and therapeutic agents to solid tumors by pHLIP peptides

**DOI:** 10.3389/fphar.2024.1355893

**Published:** 2024-03-13

**Authors:** Yana K. Reshetnyak, Oleg A. Andreev, Donald M. Engelman

**Affiliations:** ^1^ Physics Department, University of Rhode Island, Kingston, RI, United States; ^2^ Molecular Biophysics and Biochemistry Department, Yale, New Haven, CT, United States

**Keywords:** pHLIP targeting of cell acidity, cell acidity as a biomarker, targeting diverse tumor cells, immune therapy strategies, targeted nanoparticle delivery, tumor imaging and diagnosis, targeted drug delivery, targeted delivery to the cytoplasm

## Abstract

The family of pH (Low) Insertion Peptides (pHLIP) comprises a tumor-agnostic technology that uses the low pH (or high acidity) at the surfaces of cells within the tumor microenvironment (TME) as a targeted biomarker. pHLIPs can be used for extracellular and intracellular delivery of a variety of imaging and therapeutic payloads. Unlike therapeutic delivery targeted to specific receptors on the surfaces of particular cells, pHLIP targets cancer, stromal and some immune cells all at once. Since the TME exhibits complex cellular crosstalk interactions, simultaneous targeting and delivery to different cell types leads to a significant synergistic effect for many agents. pHLIPs can also be positioned on the surfaces of various nanoparticles (NPs) for the targeted intracellular delivery of encapsulated payloads. The pHLIP technology is currently advancing in pre-clinical and clinical applications for tumor imaging and treatment.

## 1 Tumor targeting

Targeted delivery of cytotoxic and immuno-activating agents has emerged as a highly advantageous approach to treating tumors. Targeting enhances the therapeutic index (TI) to provoke immune activation and/or killing of cancer cells within the TME while attenuating systemic toxicity and immuno-activation. Successful targeting strategies have included the uses of antibodies where suitable biomarkers are particularly abundant in a tumor, or the use of specific ligands where binding proteins are enriched on tumor cell surfaces. Approaches using these strategies have extended lives and relieved symptoms, but the long-term benefits have been challenged by the selection of resistant tumor cell clones, resulting in tumor resurgence. In the present discussion, we consider the targeting of a general biomarker that is present as a consequence of the highly active metabolism in the TME: cell surface acidosis.

### 1.1 Acidity of the tumor microenvironment

Highly proliferative cancer cells, tumor stroma and activated immune cells within the TME tend to employ enhanced glycolysis in response to high energy demands, either in the presence of oxygen (Warburg effect) or in hypoxic conditions (Pasteur effect) significantly acidifying the extracellular space (Warburg et al., 1927; Krebs, 1972; Damaghi et al., 2013; Swietach et al., 2014). Also, cancer cells located next to the stroma, primarily consisting of cancer-associated fibroblasts (CAFs), tumor-associated macrophages (TAMs), myeloid derived suppressor cells (mMDSCs) and regulatory Treg cells, can consume lactate and other metabolites promoting oxidative phosphorylation (OXPHOS), a phenomenon known as the Reverse Warburg effect (Wilde et al., 2017). This metabolite flow allows a “crosstalk” between cancer and stroma cells, which leads to tumor expansion and triggers the development of “cold” tumor phenotypes characterized by excluded or impaired cytotoxic T- and NK-cells (Mantovani et al., 2017; Wu et al., 2020; Tu et al., 2021). The main byproduct of OXPHOS is carbon dioxide (CO_2_), which can freely diffuse across membranes along its concentration gradient. In the extracellular space, the membrane-bound enzyme carbonic anhydrase 9 (CAIX), which is overexpressed in many tumors, catalyzes the hydration of CO_2_ to produce protons (H^+^) and bicarbonate (HCO^−3^) ions, contributing to acidification of the extracellular space (Swietach et al., 2007). Thus, either overactivated glycolysis or OXPHOS can lead to an excess of protons (H^+^) around metabolically active cells.

### 1.2 Cell surface acidity

Acidity is the highest (or pH is the lowest) at the cell surface. The flux of exported acidity lowers the pH surrounding a cell, and the proton concentration is accentuated near the cell surface both by flux and by the membrane electrochemical potential. As a result, extracellular pH (pH_e_) is lowest at the surfaces of diseased cells, where it is significantly lower than normal physiological pH or the typical bulk extracellular tumor pH (Anderson et al., 2016; Ohgaki et al., 2017; Podder et al., 2021). The low pH region persists at cell surfaces even in well-perfused areas within a diseased tissue (Gillies et al., 2012). The acidity on cell surfaces is a targetable characteristic that is not subject to clonal selection, and the level of acidity is a predictor of disease progression, with cells in more aggressive tumors being more acidic than in less aggressive ones (Estrella et al., 2013). Studies over the last few decades have demonstrated that the intracellular pH (pH_i_) of solid tumors is maintained within a range of pH_i_ ∼ 7.2–7.4, whereas the extracellular pH is acidic, i.e., pH_e_ < 6.5 (Gillies et al., 2002; Swietach et al., 2007; Zhang et al., 2010; Chen and Pagel, 2015). Additionally, it has been shown that pH at the surface of metabolically active cells is pH_surf_ < 6.0 (Krahling et al., 2009; Anderson et al., 2016; Ohgaki et al., 2017; Wei et al., 2019).

**FIGURE 1:**
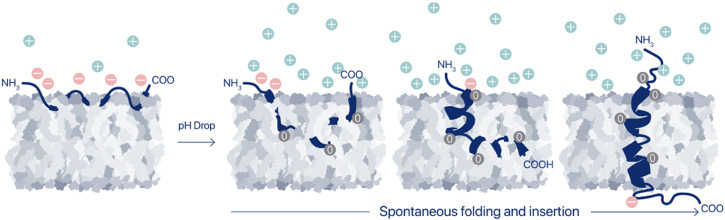
Membrane-associated folding of pHLIP. A pHLIP peptide is reversibly associated with a membrane lipid bilayer in normal (healthy) tissues, where the extracellular pH is in the range of 7.2–7.4 (state II). Asp and Glu residues in the pHLIP sequence carry negative changes, which prevent propagation of the pHLIP into the hydrophobic layer of the membrane at normal and high pHs, and the binding is readily reversible. Thus, peptides are eventually washed out of normal tissues *in vivo*. However, when the extracellular pH, and specifically, the cell surface pH (pH_surf_) is low, it triggers the protonation of the negatively charged carboxyl groups, which in turn leads to an increase of the peptide’s hydrophobicity, leading to insertion and folding (coil-helix transition) within the membrane and culminating by transmembrane helical orientations of pHLIP (state III).

Thus, agents that can sense pH at the surfaces of cells may achieve high sensitivity and specificity. pHLIPs are a family of moderately hydrophobic peptides with a modest affinity for cell plasma membranes at normal pH. When they are at a cell surface, they respond to the surface pH_surf_ and, if the pH is acidic, they insert into the membrane to form stable transmembrane (TM) helices, typically with the C terminus positioned in the cytoplasm and the N terminus remaining in the extracellular space (Figure 1). Any protonated carboxyl groups on the inserting end of the peptide are translocated in their neutral form across membrane into the cytoplasm. Since the pH in the cytoplasm is nearly neutral; and de-protonation of carboxyl groups occurs, they become negatively charged. These charges help to serve as anchors for pHLIP peptides in the membrane, significantly reducing the exit rates of pHLIP’s from the cellular membranes (Barrera et al., 2011; Karabadzhak et al., 2012; Weerakkody et al., 2013; Demoin et al., 2016).

### 1.3 Discovery of pHLIP

The first pH-sensitive peptide of the pHLIP family was discovered by John Hunt during his Ph.D. work with Engelman in 1993–1997, in the course of fundamental studies directed toward understanding membrane protein folding. Seven polypeptides corresponding to the alpha helices of the membrane protein bacteriorhodopsin (BR) were synthesized, and the structure of each individual polypeptide was studied in phospholipid vesicles to test the then-current idea that they would be independently stable in a membrane (Hunt et al., 1997a). It was found that a 36-amino acid polypeptide containing the C helix of BR (BRC peptide) spontaneously equilibrates in a pH-dependent manner between a TM alpha-helical conformation (state III), a peripherally bound nonhelical conformation (state II), and a fully water soluble conformation (state I), with the transmembrane form predominating at low pH (Hunt et al., 1997b). Insertion of the BRC peptide into the lipid bilayer occurred with a *pK* of 6.0, and the process was rapid and fully reversible. The phenomenon was interesting, and it was felt that there might be practical applications, but it was only 10 years later, in 2003–2006, that Drs. Reshetnyak, Andreev and Engelman continued the investigation to measure thermodynamic and kinetic parameters and to explore more details of the molecular mechanism of bilayer interactions (Reshetnyak et al., 2007; Reshetnyak et al., 2008; Tang and Gai, 2008; Zoonens et al., 2008; Andreev et al., 2010). At that time the BRC peptide was renamed as pHLIP (specifically, it was WT pHLIP, for Wild Type), and the first biomedical applications of the pHLIP technology were pursued (Reshetnyak et al., 2006; Andreev et al., 2007; Andreev et al., 2009; Segala et al., 2009; Vavere et al., 2009).

### 1.4 Molecular mechanisms of pHLIP action

The mechanism of the pH-triggered membrane-associated folding of pHLIPs has been investigated in detail using single tryptophan pHLIP variants, which allowed to follow propagation of the different parts of the peptides into and across the membrane lipid bilayer, the process associated with coil-helix transition (Karabadzhak et al., 2012; Slaybaugh et al., 2020). It was shown that the equilibrium is shifted from coil to helical structures as the peptide partitions deeper into the hydrophobic region (Figure 1). The activation barrier for membrane insertion increases (by orders of magnitude) with an increase of the number of protonatable groups in the peptide sequence and with the presence of polar or charged payloads at the membrane-inserting end of the peptides (Karabadzhak et al., 2012; Karabadzhak et al., 2018). Also, the membrane lipid composition and the presence of ions have been shown to modulate membrane-associated folding (Barrera et al., 2012; Kyrychenko et al., 2015; Narayanan et al., 2016; Wyatt et al., 2018; Karabadzhak et al., 2018; Vasquez-Montes et al., 2018; Schlebach, 2019; Scott et al., 2019; Westerfield et al., 2019; Vasquez-Montes et al., 2022a; Vasquez-Montes et al., 2022b). The sequence of the original WT pHLIP was varied, and a family of pHLIP peptides was designed and investigated (Musial-Siwek et al., 2010; Barrera et al., 2011; Fendos et al., 2013; Weerakkody et al., 2013; Nguyen et al., 2015; Onyango et al., 2015; Deskeuvre et al., 2022; Silva et al., 2023; Zong et al., 2023). While there are interesting influences of other factors, it seems clear that the pronation of Asp and Glu residues, or their non-canonical analogs, located in the peptide’s membrane-inserting TM and flanking sequences, play key roles in the pH-triggered membrane-associated folding/unfolding. These protonation/deprotonation events frame the biomedical relevance and applications of the pHLIP technology. The *pKa* of membrane insertion is determined by the *pKa*’s of protonation of individual carboxyl groups, and their *pKa* shifts to higher pH values with the lowered dielectric constant as the peptide propagates into the hydrophobic core of the lipid bilayer, where water is scarce (Vila-Vicosa et al., 2018; Silva et al., 2021; Silva et al., 2022; Silva et al., 2023). pHLIP peptides of different sequences without and with payloads attached to one or both of the termini may have very different configurations and bilayer affinities in state II (adsorbed by the membrane at neutral or high pHs), ranging from loosely bound and mainly unstructured to partially folded and embedded deeper in the bilayer. However, in most cases, the membrane-inserted states of pHLIPs (state III) are similar due to the formation of main chain H-bonds, which drives similar helical backbone conformations. Since starting positions in the folding pathways (or membrane-associated coil-helix transitions) are different for different pHLIPs, the thermodynamic and kinetics parameters could vary considerably. Some investigators have observed intermediate states in the pathway of folding for particular pHLIP peptides (Otieno et al., 2018; Vasquez-Montes et al., 2018; Otieno and Qiang, 2021). The presence and existence of observable intermediate states is largely dependent on many variables, including pHLIP’s sequence, the lipid composition, bilayer asymmetry, ionic strength, and the nature of any payloads. However, pHLIP peptides with a minimum number of protonatable groups and a truncated membrane-inserting end undergo an apparent all-or-none transition for membrane insertion and helix formation (Karabadzhak et al., 2012). Employing the approaches of statistical physics, free-energy landscapes were constructed for pHLIP in membranes at high and low pHs, describing pHLIP’s states (Sharma et al., 2015; Sharma et al., 2022). Two viewpoints seem essential: first, the statistical ensemble of different states should always be considered, so one is viewing a predominant subset and not a single state, and second, the kinetics is especially important for *in vivo* applications in systems with a constant and fast blood flow. From a clinical perspective, the variables that count are those that modulate the behaviors of clinical lead compounds in cells, where the complex, asymmetric membranes, pH gradient and compositional environments set a stage that is not represented by the simple model systems. The complexity is significantly greater in tumors, with added variables arising from mutual cell interactions and influences from the animal host. Thus, the observations in simple systems serve as helpful guides, but cannot fully define the choices needed for the clinical uses of pHLIP.

### 1.5 pHLIPs target the TME

Much has been learned about possible clinical applications from attaching reporter molecules to pHLIP and using their signals to study tumor targeting *in vivo*. A minimal perturbation is expected from attaching probes to the end of a pHLIP that remains outside the membrane after insertion, and kinetic influences, but not major equilibrium influences may arise from positioning probes on the end that inserts across. Influences may also arise given the complexity of interactions in an animal or in intact tissue. However, the consistency of results across many variations of composition, kinds of tumor, varieties of administration, and variations in constructs leads to a well-supported view that tumors are successfully targeted *in vivo*.

Many different fluorescent dyes (Andreev et al., 2007; Reshetnyak et al., 2011; Adochite et al., 2014; Cruz-Monserrate et al., 2014; Karabadzhak et al., 2014; Tapmeier et al., 2015; Adochite et al., 2016; Miska et al., 2021; Visca et al., 2022) and positron emission tomography (PET) and single photon emission computed tomography (SPECT) imaging agents (Vavere et al., 2009; Daumar et al., 2012; Macholl et al., 2012; Viola-Villegas et al., 2014; Demoin et al., 2016; Yu et al., 2021; Chen et al., 2022; Wu et al., 2022) have been conjugated with different pHLIPs to observe tumor-targeting. Routes of administration have included intraperitoneal, intravenous, retro-orbital and sub-cutaneous injections. pHLIP uptake has been followed in more than 20 different animal tumor models including transgenic mouse models (Reshetnyak et al., 2011; Adochite et al., 2014; Cruz-Monserrate et al., 2014; Cheng et al., 2015; Tapmeier et al., 2015) and human cancer tissue specimens (Loja et al., 2013; Luo et al., 2014; Golijanin et al., 2016; Brito et al., 2020; Mitrou et al., 2021).

A variety of results support the idea that targeting *in vivo* is based on tumor acidity. Targeting has been shown to be positively correlated with tumor extracellular pH (Vavere et al., 2009; Macholl et al., 2012; Tapmeier et al., 2015), and it is enhanced by acidification using co-injection of glucose (Reshetnyak et al., 2011) or overexpression of CAIX (Tapmeier et al., 2015). Conversely, tumor targeting has been shown to be reduced by alkalization of tumors in mice fed with bicarbonate drinking water (Rohani et al., 2019). In multiple studies, it was found that non-protonatable (at low pH) pHLIP variants, where some or all Asp/Glu residues were replaced by Lys, did not target tumors and did not exhibit pH-dependent membrane insertion (Andreev et al., 2007; Reshetnyak et al., 2011; Cruz-Monserrate et al., 2014; Wyatt et al., 2018). Distributions of pHLIP peptides within tumors and their correlation with a variety of markers of tumor aggressiveness and invasiveness including CAIX, lactate dehydrogenase (LDH), Ki67 nuclear protein, and matrix metalloproteinases 7 (MMP7) have been demonstrated (Adochite et al., 2014; Rohani et al., 2019). Acidic regions targeted by pHLIP were not restricted to hypoxic areas. Highly proliferative, invasive regions at the tumor-stroma interface are very well marked by pHLIP peptides (Rohani et al., 2019; Gillies, 2021; Moshnikova et al., 2022a). Within the TME, cancer cells, CAFs, TAMs and mMDSC are marked by pHLIP (Sahraei et al., 2019; Moshnikova et al., 2022a; Visca et al., 2022). In addition to primary tumors, satellites near the primary tumor, pre-metastatic niche and micro-metastases in distant organs have been shown to be well targeted by pHLIP peptides (Segala et al., 2009; Reshetnyak et al., 2011; Cruz-Monserrate et al., 2014; Adochite et al., 2016; Rohani et al., 2019; Crawford et al., 2020; Gillies, 2021; Matsui et al., 2023).

Currently, pHLIP-based imaging and therapeutic agents are advancing in clinical trials, and more agents are in the process of clinical translation. The straightforward interpretation of a large and growing body of diverse evidence is that pHLIPs target different types of cells in tumors based on their surface acidity, and that they are a promising tumor-agnostic targeted delivery approach for the imaging and treatment of tumors.

## 2 Extracellular delivery of imaging and therapeutic payloads by pHLIP

**FIGURE 2:**
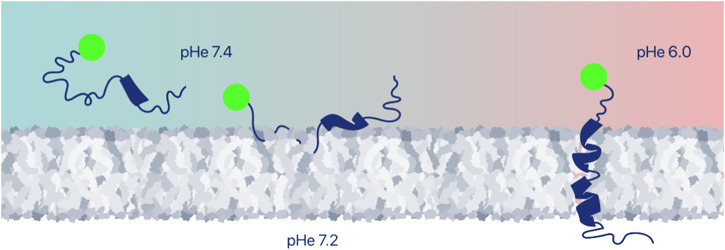
Extracellular delivery of imaging or therapeutic payloads. Various payloads can be positioned at cell surfaces within tumors by conjugation to the pHLIP terminus that remains outside of the cell. Among the useful payloads are fluorescent dyes, PET and SPECT imaging agents, various antigens, or immune-cell-recruiting molecules, and proteins. Peptide and protein payloads might be expressed with pHLIP as a single polypeptide.

When a pHLIP peptide inserts into a cell membrane, it spans the membrane lipid bilayer, positioning one terminus (typically the N-terminus) in the extracellular space and the other terminus (typically the C-terminus) in the intracellular space, creating an opportunity for targeted delivery of cargoes either to the outside or to the inside surfaces of the membrane by conjugation to one or the other terminus. Imaging and immuno-activating agents can be conjugated via non-cleavable links or expressed as fusion proteins together with pHLIP for extracellular delivery (Figure 2). It is important to note that cells eventually take up these extracellularly delivered payloads by endocytosis. This intracellular uptake does not influence most imaging applications, while it may limit the immuno-activating function of immuno-stimulating agents. Targeted cell surface delivery offers a wide range of applications.

### 2.1 pHLIP-ICG imaging agent for fluorescence-guided surgery

pHLIP-ICG is a novel imaging agent, where indocyanine green (ICG) is a near infrared (NIR) fluorescent dye chosen for clinical development with pHLIP, since ICG is FDA-approved and widely used for blood flow imaging (Crawford et al., 2020). Importantly, the fluorescence of pHLIP-ICG is enhanced about 20-fold when tethered to a cellular membrane when compared with the emission of pHLIP-ICG in aqueous solution (Golijanin et al., 2016; Roberts et al., 2019; Crawford et al., 2020). pHLIP-ICG was used to image tumors in various mouse tumor models (Crawford et al., 2020; Moshnikova et al., 2022a; Visca et al., 2022) and *ex-vivo* in human bladder and upper urinary tract specimens using different clinical instruments including da Vinci (Golijanin et al., 2016) and Stryker endoscopic systems (Brito et al., 2020; Moshnikova et al., 2022b). Compared to white light, NIR fluorescence pHLIP-ICG imaging evaluation had a higher sensitivity of 98% vs. 81% in the detection of malignant lesions in human bladders (Moshnikova et al., 2022b). The sensitivity of pHLIP-ICG imaging was 100% compared to 79% for white light examination in human upper urinary tract specimens (Brito et al., 2020). Currently, pHLIP-ICG agent is tested in fluorescence-guided surgery clinical trials on breast cancer patients in combination with Stryker SPI-PHY clinical imaging device (NCT05130801). Due to the tumor-agnostic nature of the agent it will be tested on other tumors as well. pHLIP-ICG has the potential to decrease the rate of positive surgical margins. The utility of pHLIP-ICG was also demonstrated in opto-acoustic and short-wave infrared (SWIR) imaging (Roberts et al., 2019; Mc Larney et al., 2023).

### 2.2 pHLIP-Zr for PET imaging of TME

Quantitative whole-body imaging using PET has proven to be very useful in visualizing cancer lesions. The acidic TME can be imaged with pHLIP-based PET-compatible radiotracers and the following radiotracers were evaluated ^18^F, ^99m^Tc, ^64^Cu, [^18^F]AlF, and ^68/67^Ga with pHLIP peptides in various tumor models (Vavere et al., 2009; Daumar et al., 2012; Macholl et al., 2012; Viola-Villegas et al., 2014; Demoin et al., 2016; Pereira et al., 2020). A PET imaging agent, pHLIP-^18^F, was evaluated in a phase I microdose study. The agent was found to be safe, no-adverse events were observed and tumor targeting was established. However, even at 4 h post-injection, a significant amount of the signal was observed as high background in the blood and major organs. To improve contrast, the longer-lived PET radionuclide zirconium-89 (^89^Zr) (half-life of 3.3 days) was bound to pHLIP (Bauer et al., 2022). ^89^Zr’s relatively low positron energy (E_Avg_ = 395 keV) allows for high-resolution PET imaging, comparable to ^18^F (Lee et al., 2015). A straightforward chemical conjugation route was introduced and six Zr-radiolabeled pHLIP imaging agents were tested in tumors in animal models, resulting in a candidate for clinical translation. The biological blood clearance half-life was 16.0 ± 0.4 h, and the biological half-life for total body excretion was 415 ± 10 h. Optimal tumor uptake was detected at the 48-h timepoint (9.7 ± 1.7 %ID/g). The pHLIP agent was present in the entire tumor mass, and the highest activity areas overlapped with the tumor stroma. Preclinical biodistribution results, together with human dosimetry estimates, suggest that pHLIP-Zr is expected to be safe and effective at the administered activities required to obtain diagnostic quality PET images in patients. pHLIP-Zr is expected to be a first-in-class PET agent for imaging of TME acidity. It could be used to assess the metabolic status of tumors, enabling prediction of the responsiveness to therapy by pHLIP-based therapeutics and a variety of immune therapies.

### 2.3 Antigen-pHLIP immuno-activating therapeutic agents

The idea of targeting immunogenic epitopes to cancer cells to promote immunological responses or cytotoxic activity has been recognized as an attractive approach to tumor therapy. One of the widely investigated immunogenic epitopes is α-Gal (Galα1,3Galα1,4GlcNAc-R), since humans exhibit specific anti-Gal reactivity. Humans possess anti-Gal antibodies (∼1% of immunoglobulins), and these antibodies are responsible for the strong allergic reaction triggered during organ transplantation from animals to humans (xenotransplantation), since the α-Gal epitope induces complement-dependent cytotoxicity (CDC) and antibody-dependent cell-mediated cytotoxicity (ADCC) leading to organ rejection (Larsen et al., 1990; Commins and Platts-Mills, 2013; Hamanova et al., 2015; Cooper, 2016). Therefore, the α-Gal epitope was being developed for decoration of cancer cells to induce immune attack and “tumor rejection” (Macher and Galili, 2008; Tanemura et al., 2013; Huai et al., 2016; Anraku et al., 2017). In clinical trials with the α-Gal epitope, lipids were used to position α-Gal at the surfaces of cancer cells via intra-tumoral administration of α-Gal-lipid (Whalen et al., 2012; Albertini et al., 2016), but the agent is not suited for systemic administration. Systemic administration of the α-Gal epitope in animals was achieved using pHLIP targeted delivery (DuPont et al., 2022). The α-Gal epitope was conjugated to a pHLIP peptide via a polyethylene glycol (PEG) linker to obtain Gal-pHLIP, and therapeutic efficacy was tested in immunized A3galt2 knockout mice, lacking α-Gal epitopes, and using a B16F10 melanoma tumor model (Thall et al., 1995; Porubsky et al., 2007). The treatment led to a reduction of tumor growth by 66% (DuPont et al., 2022).

The repertoire of immunogenic epitopes could be significantly enhanced if therapeutic efficacy did not rely on the presence of natural antibodies, but instead relied on the production of anti-epitope endogenous antibodies induced by immunization against the selected epitope. Therefore, several HA-pHLIP agents were designed and tested (DuPont et al., 2022). The HA peptide (YPYDVPDYA) from the influenza virus was selected as a highly immunogenic exogenous epitope, since it is absent in the human genome (Wilson et al., 1984), and since a high titer of anti-HA antibodies is developed in mice after immunization with KLH-HA (DuPont et al., 2022). HA peptides were attached to the N-terminal sequence of pHLIPs either directly or via PEG polymers. To enhance the overall binding affinity of HA-pHLIP to anti-HA antibodies, double headed HA-pHLIP agents were introduced, where two HA epitopes were linked to a single pHLIP peptide via PEG12 polymers. The pHLIP sequence was modified to compensate for the addition of polar HA peptides and PEG polymers and to ensure the proper *pK*s (in the range of pH 5–6) for insertion of the entire construct into membranes. The HA epitope was targeted to cancer cells by pHLIP in a mouse tumor model, and it remained exposed to the extracelullar space within tumors for about 24 h. Treatments of imunized mice, bearing 4T1 triple negative or B16F10 MHC-I negative melanoma tumors, with a double-headed HA-pHLIP resulted in a 55% reduction of tumor growth. Further reduction of tumor growth was problematic to achive, since all of the anti-HA antibodies in the blood were captured after 3 injections of HA-pHLIP and additional boosts would be required to restore the level of free anti-HA antibodies to potentially induce a more significant therapeutic efficacy.

In addition to the targeted delivery of antigens, the surface display of cancer cell molecules that promote the recruitment of immune cells was investigated (Sikorski et al., 2022). pHLIP was conjugated with a formyl peptide receptor ligand (FPRL) to form FPRL-pHLIP agent. FPRL interacts with the N-formyl peptide receptor (FPR) primarily expressed in phagocytic leukocytes (neutrophils, monocytes, dendritic cells, and natural killer cells). It was shown that FPRL-pHLIP activates FPR and enhances recruitment of immune cells and their tethering to cancer cells, which is expected to trigger an immune response.

As mentioned above, a challenge for the immune display approach is the turnover of the cell surface via endocytosis. The time needed for intracellular uptake depends on the type and size of a payload and type of targeted cells. If phagocytotic cells, such as macrophages, are targeted, rapid intracellular uptake will be observed, while antigens could be exposed longer to the extracellular space within tumors (about 24 h) when cancer cells are targeted (DuPont et al., 2022; Frolova et al., 2022; Visca et al., 2022). Nevertheless, the targeted extracellular delivery of antigens and immune stimulating molecules by pHLIPs opens opportunities to overcome the limitation of tumor antigen heterogeneity, broadening the applications of NK cell immunotherapy for tumors.

### 2.4 Protein-pHLIP fusion therapeutic agents

Since pHLIP is a polypeptide, it can be expressed as a fusion with a protein, potentially targeting the protein to tumors. Fusion of WT-pHLIP with green fluorescent protein (GFP) (GFP-pHLIP) was studied in HeLa cancer cells for cellular uptake (Frolova et al., 2022). However, the first functional example of a fusion agent is a tTF-pHLIP fusion protein, where the N-terminus of pHLIP was fused to the C-terminal region of coagulation-inducing truncated tissue factor (tTF) protein (Li et al., 2015). pHLIP targeted tTF to tumors and positioned it at the surfaces of tumor cells. tTF anchored to cellular membrane triggered a coagulation cascade, which resulted in a reduction of tumor perfusion and promoted tumor regression. Another example is an Fc fragment fusion with pHLIP (Fc-pHLIP) (Ji et al., 2019). The membrane inserted pHLIP-Fc fragments efficiently activated NK cells, initiating ADCC, which led to the death of cancer cells, including antigen-negative cells. Therapeutic efficacy was demonstrated on both primary solid tumors and tumor metastasis.

A more complex fusion protein was tested consisting of 3 components: i) CD19 for targeting of anti-CD19 chimeric antigen receptor (CAR) T-cells (CAR-T) (FDA approved therapy); ii) an extracellular domain of junctional adhesion molecule (JAM) proteins that play a key role in assembly of the tight junctions and control cell-cell adhesion; and iii) pHLIP peptide (Mendoza and Mizrachi, 2022). JAMs are members of the immunoglobulin superfamily that act as barriers in controlling the permeability of the paracellular space, responsible for compartmentalization of the cellular environment and the separation of tissues (Ebnet, 2017; Steinbacher et al., 2018). pHLIP targets the fusion protein CD19-JAM-pHLIP to tumors and inserts into cancer cell membranes. JAM binds to other JAM proteins in the existing cell–cell interactions, allowing homotypic or heterotypic interactions to occur, and may also establish *de novo* cell–cell interactions, thus preventing and restraining weakly interacting cells from metastasizing. As a result, treatment with CD19-JAM-pHLIP led to a decrease of cancer cell proliferation and metastasis. The CD19 part of the fusion protein may attract anti-CD19 CAR-T cells, which will induce cancer cell eradication.

Targeted delivery of chemokines and cytokines has also attracted attention and has been the subject of many studies. For example, interleukin 2 (IL-2) plays a fundamental role both in immune activation and tolerance, since IL-2 signaling is a key contributor to downstream T cell fate through activation of different transcription factor programs (Ross and Cantrell, 2018). The ability of IL-2 to mediate tumor regression led to FDA approval for its use in the treatment of metastatic renal cell carcinoma and metastatic melanoma in the 1990s. However, the therapeutic efficacy was modest, while a wide array of side effects ranging from flu-like symptoms to life-threatening conditions such as vascular leak syndrome was reported, and so significant efforts were devoted to targeted activation or delivery of IL-2 to the tumor or secondary lymphoid tissue (MacDonald et al., 2021). Recently, a fusion protein of IL-2 and pHLIP (IL2-pHLIP) demonstrated tumor targeting and resulted in effective reduction of breast and melanoma tumors in animal tumor models (Chu et al., 2023). Another example of targeted delivery of chemokines is a fusion of chemokine C-C motif ligand 21 (CCL21) with pHLIP (Li et al., 2023). CCL21 binds to the CCR7 cell-surface chemokine receptor found on leukocytes (Yoshida et al., 1998). A fusion of CCL21 with pHLIP was performed with and without a thioredoxin (Trx) tag to obtain CCL21-pHLIP and Trx-CCL21-pHLIP. The yield of expression was high and both fusion proteins were displayed on cancer cell surfaces at low pH, where they successfully recruited CCR7-positive cells.

Recently, pHLIP was fused with the SOPP3 mutant of a singlet oxygen generating protein (miniSOG). The SOPP3 mutant of miniSOG is an effective light-driven single oxygen generator (Westberg et al., 2017). The miniSOG-pHLIP fusion agent selectively bound HeLa cells at pH < 6.8 and induced cell death after exposure to light (Frolova et al., 2023).

It seems that many pHLIP fusion constructs have promising characteristics. These constructs may prove to be of clinical value in the future.

## 3 Intracellular delivery of therapeutic payloads by pHLIP

Over several decades, a vast variety of small molecule agents have been developed to target key functions inside tumor cells, with the aim of having some selective inhibitory effect between normal and diseased tissue. Inevitably, these agents have had problematical side effects that attend their uses, giving rise to a focus on means to enhance selective targeting. Selective targeting of tumor cells presents two basic problems: targeting and delivery. For example, Antibody-Drug Conjugates (ADCs) have had successes in the clinic by targeting specific epitopes that are abundant in certain tumors, and delivering by endocytic uptake followed by lysosomal degradation and release of the agent. As mentioned above, clonal selection and limited availability of suitable biomarkers are imposing limits on the success of ADCs. pHLIP peptides may provide a viable alternative, since they target acidity, a general feature of cells in most tumors, and can deliver small molecules directly to the cytoplasm, bypassing endocytosis.

**FIGURE 3:**
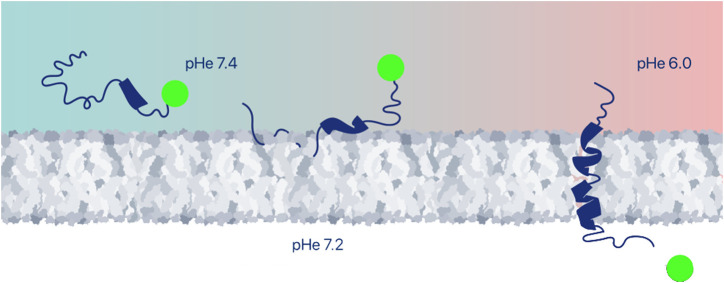
Intracellular delivery of therapeutic payloads. A large variety of payloads can be delivered across the plasma membrane into the cytoplasm of an acidic cell, and either positioned at the inner surface of the plasma membrane or released to find intracellular targets, for example, by conjugation via a cleavable disulfide bond. Among the many payloads discussed in this review are imaging agents, drugs, peptides, proteins, PNAs and siRNAs.

Many examples of successful intracellular delivery of polar and moderately hydrophobic (drug-like) payloads by pHLIP have been reported. In most cases, the payloads are conjugated to pHLIPs via disulfide linkers to the membrane-inserting end of pHLIP peptides (Figure 3). These linkers are stable outside of cells, but they are cleaved in the reducing environment of the cytoplasm, releasing the cargo. The linkers may be self-immolating, releasing the cargo in its original form—an important advantage. In early work, the intracellular delivery of fluorescent molecules and model cyclic peptides was studied to tune properties of payloads in a systematic manner and to probe the feasible ranges of delivery pHs (Reshetnyak et al., 2006; Thevenin et al., 2009), followed by the many studies of intracellular delivery of functional immuno-stimulating, cytotoxic or cell-regulating molecules described below.

### 3.1 pHLIP-STINGa for immunotherapy of solid tumors

The progression of immune-excluded “cold” tumors is associated with the formation of dense stroma consisting of acidic CAFs, TAMs and mMDSC, generating immuno-suppressive signals and impairing the homing of T and NK cells and their cytotoxic functions (Gascard and Tlsty, 2016; Sahai et al., 2020; Wu et al., 2020; Tu et al., 2021). Immuno-stimulating molecules, such as an agonist of the stimulator of interferon genes (STING) pathway (STINGa), cause the release of factors that trigger the immune response in the TME and can convert “cold” tumors into “hot” inflamed tumors (Woo et al., 2014). However, nonspecific systemic immuno-activation can be very dangerous, and the first clinical trials with small molecule STINGa have resulted in disappointingly modest efficacy (Motedayen Aval et al., 2020). Also, recent findings indicate that activation of the STING pathway in different types of cells within the TME has different benefits (Decout et al., 2021; Chamma et al., 2022). Reprogramming of M2-type TAMs (M2-TAMs) toward an M1 phenotype, suppression of CAFs and mMDSCs, as well as activation of dendritic cells (DCs) to train T cells are advantageous, while activation of T cells is associated with the development of pro-apoptotic signals. pHLIP was able to accomplish a number of desirable tasks in the delivery of STINGa: i) pHLIP-STINGa extended the lifetime of a STINGa in the blood; ii) > 70% of CAFs and M2-TAMs, and >50% of mMDSCs and DCs within TME were targeted by pHLIP-STINGa, resulting in activation of cytokines within the TME; iii) the tumor stroma was destroyed (the number of CAFs was reduced by 98%), triggering intratumoral hemorrhage, which led to an increase of pH within the TME (Moshnikova et al., 2022a). As a result, a single dose of pHLIP-STINGa administered either by intravenous or intraperitoneal injection eradicated tumors in 18 out of 20 mice, and tumors were not developed upon a re-challenge by an additional injection of cancer cells (90 days after a single dose of pHLIP-STINGa). Thus, pHLIP-targeted delivery of STINGa to tumor stroma and TAMs induces activation of signaling, potentially resulting in the recruitment and infiltration of T and NK cells, which gain access to the tumor core. The cytotoxic activity of T and NK cells was not impaired by being subjected to an acidic environment, and immune memory was developed. pHLIP-STINGa is under development for translation to clinical trials by pHLIP, Inc.

### 3.2 Cytotoxic drug payloads

Since pHLIP delivery facilitates the entry of a small molecule agent compared to diffusion of the agent by itself, the properties of a therapeutic agent can cover a larger chemical space, including more expanded criteria for polarity and molecular weight. The first polar, cell-impermeable functional payloads delivered intracellularly by pHLIP peptides include mushroom phallo- and amanita toxins (Reshetnyak et al., 2006; An et al., 2010; Wijesinghe et al., 2011; Moshnikova et al., 2013). Phalloidin is a cyclic heptapeptide cytotoxin that binds actin filaments and stabilizes them against depolymerization (Wieland, 1977). It is known that when a sufficient amount of phalloidin is microinjected into a cytoplasm, cell proliferation is inhibited (Wehland et al., 1977), but phalloidin is large and polar, and does not enter a cell on its own. WT pHLIP was shown to deliver phalloidin and phalloidin-tetramethylrhodamine (TRITC) to a variety of cancer cells in a pH-dependent manner, leading to stabilization of the actin cytoskeleton and formation of multinucleated cells, and resulting in the inhibition of cell proliferation (Reshetnyak et al., 2006; An et al., 2010). To facilitate pHLIP-mediated delivery of polar phallacidin, an analog of phalloidin, diamines of various lengths of hydrophobic chains were attached to it (Wijesinghe et al., 2011). The results indicated that translocation of polar cargoes by pHLIPs can be facilitated by hydrophobic molecular entities; however, a balance is required, since the hydrophobic facilitators can induce aggregation of a pHLIP-payload construct and reduce its affinity for a membrane. The *pK* of pHLIP-mediated intracellular delivery of amanitin was tuned by using linkers of different hydrophobicity, and cancer cell viability was assessed after exposure to the agents (Moshnikova et al., 2013). Amanitin is a highly selective allosteric inhibitor of eukaryotic polymerase II, and is one of the deadliest toxins known, exhibiting toxicity against both dividing and quiescent cells, which has made it an attractive payload for antibody drug conjugates (ADCs) (Davis and Preston, 1981; Moldenhauer et al., 2012; Nicolaou and Rigol, 2019; Li Y. et al., 2021). The cytotoxic effect was monitored when amanitin was conjugated to the pHLIP’s membrane inserting end via a S-S cleavable linker, and no cytotoxicity was observed when a non-cleavable linker was used, or when amanitin was conjugated to the membrane non-inserting end of pHLIP (Moshnikova et al., 2013). Potency, which was defined as a difference between cell viability at low and physiological pHs at different concentrations of the constructs, was enhanced by bundling 2 or 4 pHLIP-amanitin agents in a single bundle (Wyatt et al., 2018). Despite the promising data obtained on cultured cells, systemic administration of pHLIP-amanitin was associated with liver toxicity, since hepatocytes have a special transporting system for the uptake of small cyclic molecules like phallo- and amanita toxins (Munter et al., 1986), and pHLIP peptides conjugated with these toxins did not prevent uptake by liver cells (unpublished results). Recently it was shown that a 3-4x enhanced cytotoxicity of pHLIP-amanitin was observed for urothelial cells with a 17p loss after 2 h of treatment at pH6 (Moshnikova et al., 2022b). While systemic use appears unlikely to succeed, an application for bladder cancer may be possible using instillation into the bladder lumen. Based on the data so far, it appears that such a topical treatment of superficial bladder cancers with pHLIP-amanitin may be feasible.

Tubulin inhibitors constitute another group of cytotoxic compounds widely used in ADCs, including monomethyl auristatin E and F (MMAE and MMAF) and maytansinoid, ravtansine (DM4). When the tubulin protein is targeted to prevent microtubule formation, cell death by apoptosis results. The anti-CD30-MMAE (brentuximab vedotin) ADC is approved for the treatment of Hodgkin’s lymphomas. pHLIP was tested with MMAE and MMAF for inhibition of cancer cell proliferation and treatment of solid tumors in mice (Burns et al., 2015; Burns et al., 2017). pHLIP-DM4 (CBX-13) and pHLIP-MMAE (CBX-15) are under development for translation to clinical trials by Cybrexa Therapeutics. DM4 and MMAE inhibitors are linked to pHLIP’s membrane-inserting end via cleavable self-immolating linkers, and a candidate has been designated, CBX-15. The promising data presented at the 35th AACR-NCI-EORTC (ANE) Symposium in 2023 indicate that CBX-15 rapidly regressed tumors in animal models, resulting in complete responses without damaging healthy tissues such as bone marrow, and invoked an increased resistance to live tumor rechallenge and a doubling of bone marrow-resident CD4 T-cells 50 days post-dose.

A pHLIP-drug conjugate that is under clinical development by Cybrexa and Exelixis is CBX-12 or pHLIP-exatecan (NCT04902872). Exatecan is a potent DNA damaging agent that acts as an inhibitor of topoisomerase, an enzyme that regulates DNA structure by cleaving and rejoining DNA during normal cell cycle progression (Pommier et al., 2016). In multiple xenograft models pHLIP delivered exatecan to tumors, inducing tumor cell killing with minimal to no bone marrow or gastrointestinal toxicity, which are typical for free exatecan (Gayle et al., 2021). Also, pHLIP-exatecan combined with anti-PD-1 or anti-CTLA4 agents resulted in delayed tumor growth and some complete responses (Gayle et al., 2022). The agent stimulated expression of MHC-1 and PD-L1 and induced immunogenic cell death resulted in a long-term immune recognition of tumor cells and antitumor immunity, which could be associated with delivery of exatecan to stroma and activated myeloid cells within the TME.

Among other cytotoxic drugs that have been explored for intracellular delivery by pHLIP, are doxorubicin (DOX), etomoxir and calicheamicin. pHLIP-DOX was effective on both drug-sensitive and drug-resistant cells, which may allow a way to overcome the issue of multi-drug resistance (MDR) (Song et al., 2016). Also, it was shown that the dendrimeric display of DOX on the pHLIP carrier facilitates the pH-dependent release of drug to the cytosol, eliminating endosomal sequestration of the drug, and augments DOX cytotoxicity relative to the free drug (Burns and Delehanty, 2018). pHLIP-etomoxir, where etomoxir is an inhibitor of carnitine palmitoyltransferase 1, efficiently slowed down the growth of various cancers (Deskeuvre et al., 2022). Calicheamicin, a potent cell-cycle independent enediyne antibiotic that binds and cleaves DNA, has been approved in ADCs with an anti-CD33 antibody (gemtuzumab ozogamicin or Mylotarg) and an anti-CD22 antibody (inotuzumab ozogamicin or Besponsa) for the treatment of liquid cancers (Hamann et al., 2002; Ricart, 2011; de Vries et al., 2012; Kantarjian et al., 2016). pHLIP-calicheamicin was effective in inhibiting tumor growth in mice and depleting CD206^+^ TAMs within the TME.

### 3.3 Peptide and protein therapeutic payloads

pHLIPs have been used for targeted delivery of peptides and proteins either conjugated via S-S cleavable or non-cleavable bonds or expressed as single polypeptide chains with the goal of triggering cell-regulation pathways. Such constructs could be regarded as synthetic membrane receptors, with extracellular and/or intracellular domains and pHLIP as a tumor-targeting TM part of the receptor. One of the scaffolds for such a receptor was introduced using amphiphilic DNA tetrahedrons (Ts) composed of a pendent DNA and cholesterol tags (Li et al., 2019; Li et al., 2021) as an extracellular domain and protein recruiter (Pr) as an intracellular domain to locate proteins at the inner leaflet of the plasma membrane bilayer. Ts-pHLIP-Pr was tested on cells to demonstrate proof of principle (Wu et al., 2023).

In another example, a P1AP peptide (KKSRALF) that mimics i3 of the protease-activated receptor (PAR1) was conjugated to the C-terminus of different pHLIPs through a non-labile chloro-acetylchloride linker (Burns and Thevenin, 2015), cleavable S-S linker (Yu et al., 2020) or was synthesized as a single polypeptide (Chen et al., 2021). PAR1 is a member of G protein-coupled receptors (GPCRs) family and targeting of its intracellular portion modulates the interaction of GPCRs with G proteins (Covic et al., 2002). P1AP peptide, intracellularly delivered by pHLIP, was anchored to the cytosolic face of the plasma membrane to stabilize PAR1 in its nonactive conformation and effectively downregulate it signaling cascade. As a result, cytotoxicity was observed in cancer cells, and tumor targeting was confirmed by SPECT imaging using I^125^-pHLIP-P1AP (Chen et al., 2021).

A Trx-pHLIP-beclin1 construct composed of a Trx tag, pHLIP and an evolutionarily conserved motif of beclin1 was studied (Ding et al., 2018). Beclin1 phosphorylation releases the anti-apoptotic protein Bcl2 and activates the lipid kinase vacuolar protein sorting 34 (Vps34), which induces autophagosome formation (Pattingre et al., 2005). The Trx-pHLIP-beclin1 fusion protein was prepared by connecting of the N-terminus of the conserved motif of beclin1 to the C-terminus of pHLIP and linking N-terminus of pHLIP with Trx tag, highly hydrophilic and heat-stable protein with strong folding properties. Trx-pHLIP-beclin1 inhibited breast and ovarian cancer cells proliferation and induced autophagy. The antitumor efficacy was confirmed on SKOV3 xenograft tumor mouse model.

A different pathway, which was demonstrated to play an important role in the regulation of autophagy and cell death, is based on the interactions of transmembrane protein TM219 with insulin-like growth factor binding protein 3 (IGFBP3), which induces caspase 8 dependent apoptosis (Ingermann et al., 2010). The SCTT peptide (CFHPRRESHWSRTRL) of the cytoplasmic domain of TM219 was conjugated via an S-S bond to the C-terminal part of pHLIP (Joyce and Nour, 2019). A significant reduction of beclin1 phosphorylation was observed after intracellular delivery of SCTT by pHLIP in the presence of IGFBP3, indicating that SCTT inhibits TM219-IGFBP3 signaling when delivered to the cytoplasm and can block autophagy in 3D cell culture.

A cancer treatment might also be developed by inhibition of epidermal growth factor receptor (EGFR) (Yarden and Pines, 2012) by competitive binding to inhibit the association of its cytoplasmic juxtamembrane (JM) domain, which is essential for receptor dimerization and kinase function. The C-terminal end of a pHLIP peptide was fused with a JMA peptide (TLRRLLQ, residues 645–663 of JM) (Gerhart et al., 2018). JMA forms a short *α*-helix that interacts in an antiparallel manner to stabilize the asymmetric dimer (Jura et al., 2009; Red Brewer et al., 2009). pHLIP-JMA inhibited EGFR phosphorylation, and the downstream signaling resulted in cytotoxicity.

A pHLIP peptide has been used to deliver antimicrobial cationic peptides that form amphipathic α-helices when bound to negatively charged lipid membranes. (KLAKLAK)_2_ and its six peptide derivatives were conjugated to the C-terminus of pHLIP through a disulfide bond (Burns et al., 2016). The anti-proliferative effect of pHLIP–KLAKLAK obtained in cancer cells appeared to be due to the pH-selective translocation of the peptide across the plasma membrane, disulfide reduction, and the binding of KLAKLAK to the mitochondrial membrane to destabilize it. However, A caution in the interpretation is that positively charged peptides have a high affinity for negatively charged cell membranes and are readily taken up by endocytosis, so there may be more than one pathway for uptake, with pHLIP insertion into the endosome as an alternative route. Nonetheless, pHLIP delivery has enhanced the presence of the agent in the cytoplasm.

Even a large, polar protein payload, the 30 kDa glycoprotein gelonin, was successfully delivered by pHLIP to the cytoplasm (Ding et al., 2022). Gelonin, is a type I ribosome inactivating protein phytotoxin, which inhibits protein synthesis by cleaving Adenine 4,324 of the 28S ribosomal RNA, resulting in cell death (Shin et al., 2014). Gelonin is too toxic to be used in a non-targeted form. The Trx-pHLIP-gelonin fusion protein was prepared and tested *in vitro*, in cultured cells, and, *in vivo*, in mouse tumor models. Trx-pHLIP-gelonin treatment of SKOV3 ovarian solid tumors in mice was very effective with negligible toxicity.

### 3.4 Nucleotide-based therapeutic payloads

Other classes of polar, cell-impermeable therapeutic payloads that are not “drug-like” are siRNAs and peptide nucleic acids (PNAs). PNAs are less polar compared to siRNA, since they are artificial oligonucleotide mimetics with a peptide backbone that lacks the highly polar phosphates of a conventional nucleic acid (Nielsen et al., 1991; Egholm et al., 1993). A PNA forms more stable duplexes with DNA or RNA than either of the DNA or RNA homoduplexes, it is metabolically stable, and it could be used as an antisense, gene-regulation, immuno-modulating, or gene editing agent (Saarbach et al., 2019). PNA, being a membrane-impermeable molecule, needs to be delivered intracellularly. In an early pHLIP paper, pH-driven intracellular delivery of fluorescently labeled (by carboxytetramethylrhodamine, TAMRA) antisense PNA by WT pHLIP peptide was shown (Reshetnyak et al., 2006). Later, in a systematic study of WT-pHLIP delivery of TAMRA-PNAs of different lengths (sizes) including 12-mer (4.1 kDa), 16-mer (5.2 kDa), 20-mer (6.3 kDa) and 25 mer (7.7 kDa), effective pH-dependent intracellular translocation of PNAs up to 7 kDa was shown. Effective tumor-targeting was reduced for PNA cargoes greater than ∼6 kDa (Svoronos et al., 2020). Also, delivery of a modified PNA with (R)-diethylene glycol at the γ position (γPNA) was tested with pHLIP (Kaplan et al., 2020). pHLIP delivered antisense γPNA targeting protein KU80, a DNA double-strand break repair factor, to cancer cells and suppressed KU80 expression in a pH-dependent manner. Treatment of mice with pHLIP- γPNA led to knockdown of KU80 expression in tumors, which resulted in selective radiosensitization within tumors, but not in normal tissue.

pHLIP delivery of PNAs was successfully used to target micro RNAs (miR), which play important regulatory roles in many pathological processes. Targeting of miR-155 was shown in cultured cancer cells and in an animal tumor model (Cheng et al., 2015). pHLIP altered the biodistribution of PNA, preventing its liver accumulation and promoting renal clearance without affecting kidney function and histology. In a mouse model of lymphoma, pHLIP-PNA led to the inhibition of miR-155 and the suppression of metastasis while not affecting the level of lymphocytes.

pHLIP anti–miR-21 PNA was used to target miR-21 in tumor associated macrophages (Sahraei et al., 2019). mIR-21 regulates various downstream effectors and is associated with tumor pathogenesis during all stages of carcinogenesis (Feng and Tsao, 2016). As a result, tumor growth was reduced even under conditions where miR-21 expression was deficient in cancer cells.

The protein target of miR-29a is a carcinoembryonic antigen-related cell adhesion molecule 6 (CEACAM6), a glycoprotein that mediates cell–cell interactions and is involved in cell adhesion, proliferation, migration, invasion, and metastasis (Beauchemin et al., 1999; Blumenthal et al., 2005) and that is overexpressed in a wide variety of carcinomas (Blumenthal et al., 2007; Zang et al., 2017). pHLIPanti-miR-29a PNA was effective in the reduction of cell viability and inhibited tumor growth in a mouse tumor model as a monotherapy or combined with cisplatin, which reduced tumor volume by 40% (Son et al., 2023).

pHLIP was used for targeted intracellular delivery of a PNA conjugated to a cell penetrating peptide (CPP) (RRRQRRKKR). The PNA was designed to target long non-coding HOX transcript antisense RNA (HOTAIR), which is frequently overexpressed in solid tumors and correlates with chemoresistance and poor patient prognosis (Malek et al., 2014; Ozes et al., 2016). Treatment of mice harboring platinum-resistant ovarian tumor xenografts with pHLIP-CPP-PNA construct suppressed HOTAIR activity, reduced tumor formation and improved survival (Ozes et al., 2017).

pHLIP also mediated successful intracellular delivery of siRNAs (Zhao et al., 2018; Son et al., 2019). pHLIP-siRNA-CEACAM6 treatment resulted in tumor growth inhibition of up to 36%, and combined with cisplatin, up to 47% (Son et al., 2019). In another example, pHLIP-siRNA targeting cell division cycle-associated protein 1 (CDCA1), highly expressed in prostate cancer cells and human samples, was investigated. pH-dependent intracellular delivery of CDCA1-siRNA by pHLIP was demonstrated, as well as inhibition of tumor growth as monitored after pHLIP-siRNA treatment (Zhao et al., 2018).

While the regulatory pathways and roles of small RNA molecules are complex and interdependent, targeted pHLIP delivery of PNA or siRNA to suppress some of them in tumors has been shown and is a promising direction for possible future therapeutic development. Selective inhibition may also serve as an important research tool as the roles of the miRs are examined.

## 4 Targeted delivery of pHLIP-coated nanomaterials

**FIGURE 4:**
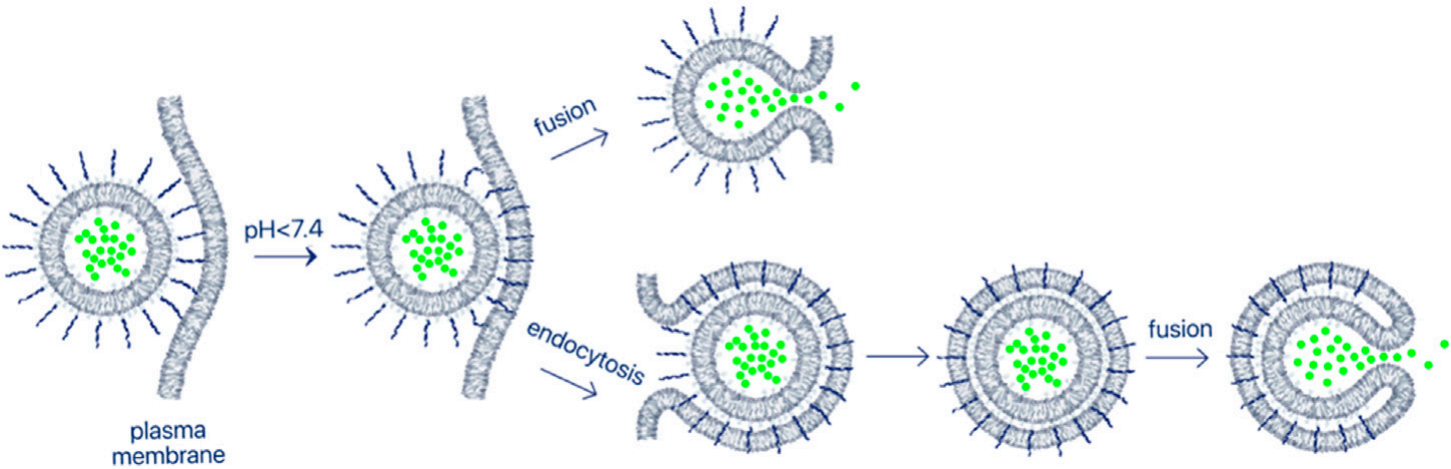
Delivery of nanomaterials. Multiple pHLIP peptides on the surface of nano-sized materials insert into the plasma membrane lipid bilayer of an acidic cell, promoting endocytotic uptake or fusion. Fusion occurs even more readily if the NP reaches the endosomal compartment, which has a very low pH. As a result, payloads encapsulated in NPs can be delivered and released in the cytoplasm. In this review, we describe targeted delivery of a large variety of NPs comprised of lipids, surfactants, metals, polymers and biological molecules coated with pHLIP.

Nanotechnology plays an important role in medicine and, specifically, in cancer imaging and treatment (Ajith et al., 2023; Sell et al., 2023). Some nanomaterials are designed for diagnostic or therapeutic applications, and most nanomaterials can also possess theranostic properties and so could be used for both imaging and therapy. The enhanced permeability and retention (EPR) effect supports passive tumor targeting of nanoparticles (NPs) due to the leaky vasculature found in some tumors. However, there are many reports indicating that active targeting or activation within the TME can significantly improve the delivery of NPs to tumors (Sun et al., 2023). pHLIP has been employed as a coating of a variety of nanomaterials for enhanced targeting and intracellular or membrane delivery of payloads. In the examples of pHLIP uses described above (Figures 2, 3), a single pHLIP peptide is conjugated with a single payload, either a small molecule or a protein. In some cases, two antigens (HA-PEG) were bound to a single pHLIP. However, when a larger entity, like a NP, is coated with multiple pHLIP peptides inserting simultaneously into a cellular membrane at low pH, cell membrane destabilization is promoted, which leads to fusion (if the NP has a lipid shell) and/or endocytotic uptake (Figure 4). An important factor to note is that pHLIP strongly inserts into the endosomal membranes, where the pH is 5.0–5.5, even more readily facilitating cytoplasmic payload release.

### 4.1 Liposomes and niosomes

In a pioneering experiment, lipid nanoparticles (LNPs) were prepared by using DOPC (1,2-dioleoyl-sn-glycero-3-phosphocholine) or DOPE (1,2-dioleoyl-sn-glycero-3-phosphoethanolamine) phospholipids and varying amounts (up to 10%) of DSPE (1,2-distearoyl-sn-glycero-3-phosphorylethanolamine) linked with 2 kDa PEG and pHLIP (Yao et al., 2013a). pHLIP promoted the fusion of the pHLIP-coated LNPs with cancer cells, resulting in intracellular delivery of a polar propidium iodine (PI) cargo encapsulated within the LNPs. The fusion also resulted in the delivery of C6 ceramide incorporated into the lipid bilayers of LNPs into the target membrane. The delivered ceramide induced cell death at low pH. Another formulation of pH-triggered fusogenic pHLIP-coated LNPs was prepared without PEG and consisted of DOPC lipids and 5% pHLIP-DSPE, with gramicidin channels included in the bilayers of the liposomes. Targeted fusion of the liposomes with a cell membrane put the channels into the membrane bilayer, leading to the disruption of the cellular monovalent ion balance and pH gradient and depolarization of the mitochondrial membrane, which resulted in inhibition of cancer cell proliferation (Wijesinghe et al., 2013).

Additionally, LNPs were prepared from DOPE, cholesterol, vitamin E, PEG-DSPE or pHLIP-DSPE, where the PEG version serves as a control for comparison with the pHLIP coated version (Wang et al., 2020). The PEG or pHLIP components were conjugated via cleavable disulfide links with DM1, a potent maytansinoid cytotoxin inhibiting the assembly of microtubules. pHLIP-LNPs and PEG-LNPs were studied in cells and mouse tumor models. When pHLIP-LNP formulations containing a few percent of fluorescently labeled lipids were used to treat cells, the fluorescent signal was found in the endoplasmic reticulum or mitochondria, as opposed to the same compositions of LNPs where pHLIP was replaced with PEG, which were mostly found in endosomes. The pHLIP-LNP formulations were more effective in inhibition of cell proliferation and tumor growth in mice.

pH-sensitive niosomes (80–90 nm in diameter) were formulated using non-ionic surfactants Span20 and Tween20, cholesterol and 5 mol% of pHLIP conjugated with DSPE lipids or the hydrophobic fluorescent dye, pyrene, which incorporates into niasome membranes (Pereira et al., 2016; Rinaldi et al., 2018). Fluorescently labeled pHLIP-coated niosomes showed tumor targeting with a resulting homogeneous distribution within tumors and a minimal accumulation in major organs. pHLIP-niosomes exhibited 2–3 times higher tumor uptake compared to the non-targeted PEG-niosomes. This lead may be worth further investigation as a delivery vehicle.

### 4.2 Gold nanomaterials

Gold NPs (GNPs) of different sizes and shapes are finding a variety of biomedical applications (Zhang and Gao, 2023). Often, GNPs are useful for the enhancement of therapeutic radiation effects. In a number of versions, the gold nanoparticles can be made to absorb near infrared radiation to produce local heating, since surrounding tissues will be relatively transparent at these wavelengths. In other versions, soft x-rays are used to produce locally effective Auger electron radiation. There have been a number of different investigtions of GNP delivery by pHLIP, with promising results.

The first example of GNP delivery employed pHLIP coated 13 nm GNPs containing europium luminescent complexes in efforts to target human platelets, which are typically not susceptible to transfection or microinjection (Davies et al., 2012). pHLIP promoted the internalization of GNPs into platelets within minutes at low pH.

Tumor targeting of GNPs functionalized with pHLIP enhanced the effectiveness of soft x-rays (20 Gray of radiation), reducing cancer cell survival and tumor size in mice through the production of Auger electrons (Yao et al., 2013b; Antosh et al., 2015; Sah et al., 2019). GNPs having a 7 nm metallic core stabilized by 0.83% wt/vol citrate, 10% pHLIP and 90% PEG were found to possess the best stability and tumor targeting (Daniels et al., 2017).

Irregular shapes, multispiked or shell-like structures of GNPs exhibit strong spatial confinement of an electromagnetic field, which leads to an increase of the excitation cross section and enhancement of plasmon polaritons (Hao et al., 2007; Melnikau et al., 2013). Disk-like shape bicelles of different aspect ratios composed of DMPC (1,2-dimyristoyl-sn-glycero-3-phosphocholine) and DHPC (1,2-dihexanoyl-sn-glycero-3-phosphocholine) lipids were used as a template for deposition of colloidal gold and coating with PEG and pHLIP, which resulted in the formation of multispiked GNPs with a mean metallic core diameter of ∼146 nm and a mean hydrodynamic size of ∼161 nm (Daniels et al., 2017). pHLIP targeting gave an excellent concentration of the nanoparticles in tumors, with little or no accumulation in muscle. The irradiation of spiked pHLIP-GNPs by an 805 nm laser led to a time- and concentration-dependent increase of local temperature that might have therapeutic potential.

Gold nanostars (GNSs) coated with pHLIPs (GNS-pHLIPs) were investigated together with non-targeting GNS-PEGs NPs as controls (Tian et al., 2017). GNS-PHLIPs exhibited higher cellular internalization at low pH compared with GNS-PEGs, and 3-fold higher breast tumor targeting in mice. GNS-pHLIPs exhibited stronger CT and photo-acoustic imaging signals compared to GNS-PEGs. Photothermal therapeutic efficacy on tumors treated with GNS-pHLIPs was observed with minimal side effects to normal tissues.

In another formulation, a photosensitizer, chlorin e6 (Ce6), and pHLIP were absorbed onto the surfaces of hollow gold nanospheres (HAuHS) to prepare HAuNS-Ce6, HAuNS-pHLIP, and HAuNS-pHLIP-Ce6 (Yu et al., 2015). HAuHS NPs (about 40 nm in size) exhibited high Ce6 and pHLIP loading capacity, forming a 4 nm shell. GNPs had a plasmonic peak in the NIR spectral range, and showed strong photothermal coupling under irradiation, which triggered the release of Ce6 and pHLIP from the surface upon heat generation. Reactive oxygen species (ROS) were produced as result of the reaction between Ce6 and surrounding oxygen in the tissues. Superior cytotoxicity, tumor targeting and photothermal effect restricted to the TME was demonstrated for HAuNS-pHLIP-Ce6 after irradiation at 670 nm or 808 nm laser wavelengths, which resulted in inhibition of tumor growth (Yu et al., 2016).

Gold nanorods were coated with mesoporous silica and capped with chitosan (CMGs) conjugated with pHLIP for multispectral optoacoustic tomography (MSOT) and drug delivery (Zeiderman et al., 2016). Treatment of cancer cells with pHLIP nanorods containing gemcitabine resulted in significantly greater cytotoxicity compared to the cytotoxicity of gemcitabine alone. pHLIP nanorods were targeted to tumors, and MSOT signal in tumors was significantly higher for pHLIP-NPs compared to non-targeted NPs without pHLIP.

A different idea is to trigger local drug release from nanocages. Gold nanocages (GNCs) were conjugated with a thermo-responsive polymer, coated with pHLIP and loaded with Doxorubicin (DOX) (Huang et al., 2019). Irradiation with a NIR laser triggers the shrinkage of the thermo-responsive polymer, resulting in the opening of the pores of the GNCs and a rapid release of DOX. DOX alone and DOX loaded into GNPs with and without pHLIP coatings were investigated in human breast MCF-7 and adriamycin-resistant (ADM) MCF-7/ADM cancer cells, in human hepatocellular carcinoma HepG2 and HepG2/ADM cells, and in MCF-7/ADM tumors in mice. The pHLIP coating significantly enhanced the cellular uptake of NPs and laser irradiation triggered the release of DOX. pHLIP-enhanced tumor targeting was observed with homogeneous distribution of released DOX within the tumor mass. The data indicate a synergistic antitumor effect and possibility a reversal of multidrug resistance.

### 4.3 Magnetic nanomaterials

By delivering atoms that have strong magnetic properties, MRI can be used to image tumors and facilitate therapies of different kinds. There have been several innovative uses of this approach, and they are described below.

In an early study, paramagnetic pHLIP-NPs were developed by using a G5–PAMAM dendrimer conjugated with about 44 chelated gadolinium atoms (Gd-DOTA-4AmP5^-^ ions), and intracellular uptake of the agent was found (Janic et al., 2016). A different Gd-based NP (AGuIX) conjugated with pHLIP were introduced for T1 MRI and radiation therapy (RT) (Liu et al., 2020). AGuIX is a 3 nm NP composed of a polysiloxane network surrounded by about 10 Gd chelates. pHLIP-AGuIX NPs were studied in cultured cancer cells and MRI was recorded in tumor-bearing mice. The use of these particles allows visualization of the tumor and establishes the concentration of the agent in the tumor and healthy tissue by MRI, which then could be used for calculation of radiation dose for treatment. Enhancement of RT occurs due to generation of a cascade of Auger electrons within the NPs.

Another approach is multimodal T1 MRI/PET using citrate-stabilized magnetic iron oxide (Fe_3_O_4_) NPs modified with PEG-pHLIP and PEG-DOTA or just PEG-DOTA, and ^68^Ga was incorporated as a chelate with DOTA (dodecane tetraacetic acid), for PET imaging (Wei et al., 2022). Both T1 weighted fast spin-echo MRI and PET signals of pHLIP-PEG-NPs in 4T1 tumors in mice were significantly higher compared to non-targeted PEG-NPs.

Manganese (II) ion (Mn^2+^), another T1 contrast agent, can be precipitated at neutral pH by arsenite trioxide (ATO) to form a manganese arsenite complex (MnAs). ATO is an FDA approved drug for the treatment of acute promyelocytic leukemia (Zhang et al., 2010). MnAs dissociates in a mildly acidic environment releasing i) Mn^2+^ ions, which enhances T1 signal, and ii) arsenite, which kills tumor (Zhao et al., 2015). NPs (30 nm in hydrodynamic diameter) were prepared where MnAs was complexed into silica shells, and the NPs were coated with fluorescent pHLIP-PEG8 to form 90 nm stable particles (Zhang et al., 2019). pH-dependent Mn^2+^ release, enhancement of T1 signal, cellular uptake, superior tumor targeting of pHLIP-coated NPs over non-targeted NPs, and significant inhibition of tumor growth and mice survival were shown.

Superparamagnetic iron oxide nanoparticles (SPIONs) of different sizes including 64, 82, 103 and 121 nm NPs were coated with pHLIPs. The 64 nm SPION-pHLIP nanoclusters exhibited the most effective pH-responsive retention in cells and gave a strong MR signal recorded in various animal tumor models (Wei et al., 2017). In other work, Fe_3_O_4_ magnetic NPs obtained by co-precipitation, modified by 3-aminopropylsilane (APS) (APS-MNP) and coated with pHLIP (pHLIP-MNP) to form NPs with 130–140 nm hydrodynamic diameters was investigated (Pershina et al., 2020a). T2 weighted MRI and distribution of Fe in breast tumors of different sizes revealed high targeting of small-to-medium sized tumors (80–400 cm^3^) and less accumulation in larger tumors (Pershina et al., 2020b). Later, similar type of MNPs coated with either PEG or PEG-pHLIP were introduced (Demin et al., 2021). A higher accumulation of pHLIP-MNP compared to PEG-MNP was shown by MRI in 4T1 breast cancer tumors orthotopically grown in BALB/c mice and MDA-MB231 xenografts. No pathological changes were noted in the organs or blood of mice after MNP administration. The amount of Fe measured within TME was directly correlated with pH measured using nanoprobe pH sensor. The areas of higher Fe accumulation had lower pH (Pershina et al., 2021).

An interesting approach was utilized by locating pHLIPs at the surfaces of *Magnetospirillum magneticum* AMB-1 magnetosomes (Schuerle et al., 2020). Magnetosomes are found in magnetotactic bacteria (MTB) that use iron to sense magnetic fields. MTB form membrane-bounded intracellular nanocrystals of magnetite (Fe_3_O_4_), which can serve as excellent T2 MRI contrast agents (Alphandery, 2014). Purified magnetosomes consisting of 40 nm NPs with single superparamagnetic domains were decorated with pHLIP peptides. pH-dependent interactions of pHLIP-coated magnetosomes were shown *in vitro*, and tumor targeting and MRI imaging were seen *in vivo* in a mouse tumor model.

### 4.4 Other metallic nanoparticles

A number of non-magnetic metals have potential for imaging or various therapeutic approaches. An imaging approach using zinc gallate (ZGO)-based persistent luminescence NPs coated with pHLIP were tested on breast cancer cells and 4T1 breast tumor targeting was demonstrated in mice (Sharma et al., 2021).

A therapeutic approach that has been discussed for years is based on the capture of an epithermal neutron by a boron atom, resulting in an alpha particle and a recoiling lithium atom, and producing very local damage. A problem has been to concentrate enough boron in a tumor. The surface of ZGO-NPs was functionalized with poly (vicinal diol), conjugated with pHLIP and loaded with large amounts of boron ^10^B for boron neutron capture therapy (BNCT) (Sharma et al., 2022). The *in vitro* evaluation of the formulation against cancer cells followed by neutron irradiation revealed its potent cytotoxicity with IC_50_ ∼ 25 μM. Animal studies performed on melanoma and fibrosarcoma tumor models gave a reduction in tumor volume (75%–80%) as compared with a control tumor after neutron irradiation.

Copper-cysteamine (Cu-Cy), a promising photosensitizing agent for photodynamic therapy (PDT) that can also be effectively activated by X-rays to produce singlet oxygen for efficient deep cancer treatment, was used to make NPs (Shrestha et al., 2019). Cu-Cy-NPs coated with pHLIP led to a reduction in tumor size in mice upon X-ray activation. Large Cu-Cy pHLIP-NPs (∼300 nm) exhibited the most intense photoluminescence, while medium sized NPs (∼100 nm) produced the most reactive oxygen species upon X-ray irradiation, and the smallest NPs (∼40 nm) gave the best outcome in the treatment of tumors in mice upon 90 kVp radiation (Sah et al., 2020).

An interesting idea is to combine a sensitizer with photothermal therapy. Hollow copper sulfide (HCuS) NPs modified with PEG-pHLIP were used for encapsulation of an inhibitor of stress granules (ISRIB) together with NIR responsive material, lauric acid (Tong et al., 2023). Stress granules regulate protein expression and cell viability under various stress conditions, and their formation is triggered by heat shock proteins, which are overexpressed in cancer cells (Mahboubi and Stochaj, 2017). As a result, the benefits of photothermal therapy (PTT) are greatly limited by the heat tolerance of cancer cells, but inhibition of stress granule formation can reduce the heat tolerance, possibly improving the therapy. The light-controlled release of the ISRIB inhibitor was found to effectively sensitize tumor cells to PTT, increasing the antitumor effect and inducing immunogenic cell death. Significant inhibition of tumor growth and of the development of lung metastases was seen, along with the infiltration of cytotoxic T-cells and reprogramming of M2-macrophages to the M1 phenotype.

### 4.5 Polymeric and silica nanoparticles

Nanoparticles based on polymer or silica technologies and targeted by pHLIP have been developed for detection, imaging and therapy of tumors. An interesting, flexible design for detection and imaging has been tested, incorporating the pHLIP technology into a multimodal nanosensor, PRISM (protease-responsive imaging sensors for malignancy), with the goal of targeting tumor acidity and using metalloproteases in the TME to release urinary reporters (Hao et al., 2021). An eight-arm PEG polymeric scaffold (40 kDa) was functionalized with pHLIP and a substrate (PLGVRGK) for matrix metalloproteinase 9 (MMP-9). Also, the pHLIP could be further modified with chelate-metal complexes for PET (or probes for fluorescence imaging), specifically ^64^Cu-NOTA was tested, and MMP-9 substrate could be modified with reporters that will be found in urine after cleavage of the substrate to serve as a detector for cancer (specifically, fluorescein amidite - biotin tags were tested). Lung metastases, which are typically challenging to image, were very well distinguished and visualized in mice using ^64^Cu-pHLIP-PRISM with a 4-fold higher signal for pHLIP-targeted PRISM compared to a non-targeted version. Importantly, the PET signal in lungs was not obscured by the signal in the heart, which is a significant issue using traditional FDG (fludeoxyglucose F18) PET imaging since there is a high cardiac uptake. Tumors derived from human colorectal cancer with low glucose uptake demonstrated an 8.6-fold pHLIP-PRISM uptake over the surrounding normal muscles, whereas the tumor uptake of FDG was indistinguishable from the background tissue signal, and progression of liver metastases was correlated with an increase of pHLIP-PRISM signal. Finally, a significant reduction of the PET signal was observed in mice after chemotherapy treatment with 5-fluorouracil/leucovorin, which indicates that pHLIP-PRISM and acidity imaging with other pHLIP agents could be used for the detection of metastases and monitoring the outcome of therapy.

Another type of NP was developed using PEG and a nitrated gluconic acid copolymer core, coated with pHLIP and loaded with the DOX prodrug, boronate-DOX (BDOX), and also the *ß*-lapachone drug (Li et al., 2021). *ß*-lapachone is a novel drug that induces the production of high level of ROS catalyzed by NAD(P)H: quinone oxidoreductase-1 (Yang et al., 2017). NPs were targeted to tumors by pHLIP and activated by high-intensity focused ultrasound. Subsequently, nitric oxide (NO) was produced by transfer of hydrophobic nitrated gluconic acid to the hydroxyl under exposure to glutathione inside a cell, followed by BDOX and *ß*-lapachone release. NPs were tested in cells and in a athymic mice breast tumor model, and significant tumor reduction with no sign of toxicity was observed as assessed by biochemical parameters.

Mesoporous silica NPs about 140 nm in diameter and with about 3 nm pores were coated with pHLIP and loaded with DOX (Zhao et al., 2013). The cellular uptake and kinetics of DOX release were investigated and cytotoxicity at low pH was monitored. Smaller (60–80 nm in diameter) thioether-bridged mesoporous organosilica NPs labeled with the fluorescent dye Cy5.5 and coated either with PEG or pHLIP, and loaded with DOX were introduced, where DOX release was responsive to glutathione. The cytotoxic effect, tumor targeting, and inhibition of tumor growth of the pHLIP-coated NPs was higher compared to PEG-coated NPs.

In another study, 26, 45 and 73 nm mesoporous silica NPs, which could be used for drug and imaging agents’ delivery, were functionalized with pHLIP (MacCuaig et al., 2021). Superior targeting of pancreatic tumors in mice was observed for pHLIP-NPs compared to non-targeted NPs, with the highest uptake of 26-nm sized pHLIP-NPs. The MSOT signal was detected from a IR780 dye retained within the NPs.

Covalent organic framework (COF) nanosheets conjugated with GNPs (COF-Au) and coated with pHLIP (pHLIP-COF-Au) were loaded with DOX and tested *in vitro* and *in vivo* (Chen et al., 2021). DOX release, cell proliferation and survival, tumor targeting and therapeutic efficacy on mice were investigated for pHLIP-coated and non-targeted NPs with or without 635 nm light illumination. The best results were achieved in cells and animals by using pHLIP-coated NPs loaded with DOX and irradiated with light.

### 4.6 Biologically-based nanomaterials

An interesting approach has been developed using a pH- and ATP-sensitive nanomaterial, where Cy5-and biotin-labeled aptamer strands are hybridized with quencher (BHQ2)-bearing complementary strands and mixed with biotin-pHLIP and streptavidin to form NPs (Di et al., 2019). In the duplex state, the fluorescence of the aptamer strand is quenched. However, ATP binding to the aptamer leads to the disruption of the duplex structure and an increase of fluorescence. ATP sensing imaging was demonstrated *in vitro* and *in vivo* in primary tumors, lung metastases, and lymph nodes. Since ATP is released from cells upon cell death, it is a marker, and the nanomaterial might find wide applications in immune-oncology studies.

An effective T-cell activation strategy was introduced by creating pHLIP-coated NPs (Qiu et al., 2018). Ovalbumin (OVA), widely used in inducing antigen-specific immune responses, and lipo-polysaccharide (LPS), an amphiphilic Toll-like receptor 4 ligand, were mixed under mild stirring and uniform spherical OVA-LPS NPs (91 nm) were formed. The NPs were coated with biocompatible and biodegradable polyphenol, a class of natural compounds abundantly found in plants and food, and that is degradable by glutathione. pHLIP was used to functionalize the NPs to facilitate endolysosomal escape and promote cytoplasmic localization, with the aim to enhance cross-presentation of the antigen by DCs to effectively activate cytotoxic T-cells. The results demonstrate that pHLIP-NPs can induce endolysosomal escape and enhance CD8 T cell activation both *in vitro* and *in vivo*.

Minicells are nanosized forms of bacteria, which can be produced in large quantities and used for drug delivery (Yu and Margolin, 2000; MacDiarmid et al., 2007). *Escherichia coli* Nissle 1917 (EcN) mainly proliferate in the interface between the necrotic and hypoxic regions of tumors and the specific cell membrane of EcN can directly interact with the adaptive immune system to reduce inflammation (Sturm et al., 2005; Stritzker et al., 2007; Arribas et al., 2009). pHLIP was expressed on the surfaces of minicells, which were then loaded with DOX (Zhang et al., 2018). In some experiments GFP was also expressed in minicells for visualization purposes. pHLIP-minicells loaded with DOX injected into mice were found in necrotic and hypoxic regions of orthotopic breast cancers, where drugs typically cannot reach due to vascular insufficiency and high interstitial fluid pressure. As a result, inhibition of tumor growth was observed.

## 5 Cancer and beyond

This review has taken its focus on pHLIP applications for the assessment, imaging and treatment of solid tumors. It is evident that there are many avenues to explore, and that some of them are progressing well in clinical trials, while others are nearing the clinical stage. The future, as has been often noted, is difficult to predict, but the rapid growth of the field and the diversity of promising approaches inspire optimism that benefits to patients are likely to emerge. The pHLIP technology may find applications not only in tumor imaging and treatment, but also in the targeting (and treatment) of inflamed and fibrotic tissues (Price et al., 2019; Visca et al., 2022; Matsui et al., 2023), atherosclerosis (Zhang et al., 2022), ischemic stroke (Ye et al., 2023) and ischemic myocardium (Sosunov et al., 2013; Hulikova et al., 2022). These diseased states are associated with elevated levels of acidity due to the presence of overactivated immune cells and/or due to hypoxia developed in diseased tissues. Time will tell!
